# TusDCB-mediated tRNA sulfur modification is required for virulence and intestinal fitness in enterohemorrhagic *Escherichia coli*

**DOI:** 10.1128/iai.00186-26

**Published:** 2026-05-15

**Authors:** Yumika Sato, Kazutomo Suzue, Hideru Obinata, Mio Horinouchi, Ayako Takita, Mai Maruhashi, Yoji A. Minamishima, Haruyoshi Tomita, Hidetada Hirakawa

**Affiliations:** 1 Department of Bacteriology, Graduate School of Medicine, Gunma University 12925, Maebashi, Gunma, Japan; 2 Department of Infectious Diseases and Host Defense, Graduate School of Medicine, Gunma University 12925, Maebashi, Gunma, Japan; 3 Core Facility Management and Technical Collaboration Center, Gunma University 12925, Maebashi, Gunma, Japan; 4 Department of Biochemistry, Graduate School of Medicine, Gunma University 38357, Maebashi, Gunma, Japan; 5 Laboratory of Bacterial Drug Resistance, Graduate School of Medicine, Gunma University 12925, Maebashi, Gunma, Japan; University of Pennsylvania School of Veterinary Medicine, Philadelphia, Pennsylvania, USA

**Keywords:** intestinal infection, bacterial pathogenesis, virulence, tRNA modification, type III secretion system, shiga-toxin producing *Escherichia coli*

## Abstract

Sulfur modification of tRNAs enhances translational accuracy and efficiency, yet its contribution to bacterial pathogenesis remains poorly understood. In *Escherichia coli*, the TusDCB complex catalyzes 2-thiolation of wobble uridines in lysine-, glutamate-, and glutamine-decoding tRNAs. To investigate its role in intestinal virulence, we constructed an in-frame *tusDCB* deletion mutant in enterohemorrhagic *Escherichia coli* (EHEC). Comparative analyzes revealed that deletion of *tusDCB* reduced transcription of type III secretion system (T3SS) genes and secretion of the T3SS translocator EspB, resulting in diminished T3SS-dependent hemolytic activity. In a *Citrobacter rodentium* mouse infection model, the *tusDCB* mutant showed attenuated colonic inflammation and reduced lethality compared with the parent strain. The mutant also displayed increased sensitivity to acid and oxidative stress, but no change in bile salt tolerance. Proteomic analysis identified decreased abundance of multiple proteins, including the T3SS translocator EspB and tryptophanase (TnaA), accompanied by loss of indole production and increased susceptibility to fosfomycin. Complementation of *tusDCB* restored these phenotypes, confirming its contribution to virulence-associated functions. Together, these findings indicate that TusDCB-mediated tRNA sulfur modification supports optimal T3SS expression, stress resistance, and bacterial fitness, thereby promoting intestinal pathogenicity in EHEC.

## INTRODUCTION

Enterohemorrhagic *Escherichia coli* (EHEC) is a well-known foodborne pathogen that causes not only severe hemorrhagic diarrhea but also life-threatening complications such as hemolytic uremic syndrome (HUS) and acute encephalopathy (1). EHEC produces two major virulence factors: Shiga toxins and effector proteins. Shiga toxins inhibit host protein synthesis, induce cell death, and are closely associated with the onset of HUS (2). Effector proteins are injected into intestinal epithelial cells via a needle-like structure known as the type III secretion system (T3SS), leading to the formation of attaching and effacing (A/E) lesions that facilitate bacterial colonization and trigger severe hemorrhagic diarrhea (3). A/E lesions are characterized by the destruction of intestinal microvilli, intimate bacterial adherence to host cells mediated by interactions between bacterial intimin and the host intimin receptor, and the formation of actin-rich pedestal-like structures beneath the attached bacteria (4). Effector proteins promote bacterial adhesion and actin accumulation, while translocator proteins such as EspB deliver these effectors into host cells (3, 5). The genes required for pedestal formation and the core structural components of the T3SS are encoded within five major operons, designated LEE1 through LEE5, collectively known as the locus of enterocyte effacement (LEE) (6). In addition to these LEE-encoded components, EHEC produces numerous non-LEE-encoded effector proteins that are located elsewhere in the genome and further contribute to host-pathogen interactions and virulence.

Treatment of EHEC infections with certain antibiotics, such as quinolones or β-lactams, can induce bacterial lysis and result in massive, transient release of Shiga toxins, thereby increasing the risk of HUS (7, 8). Consequently, antibiotic therapy is contraindicated for EHEC infections in many countries. This presents a major clinical challenge, as no definitive curative treatments or reliable preventive measures against disease progression are currently available.

TusD, TusC, and TusB are components of a sulfurtransferase complex that adds sulfur derived from cysteine to tRNA^Lys^, tRNA^Glu^, and tRNA^Gln^ (9). The *tusD* gene is transcribed as part of an operon with *tusB* and *tusC*. *In vitro* experiments using recombinant TusD, TusB, and TusC proteins have demonstrated that these proteins are involved in the sulfur modification of the uridine base at the wobble position (position 34) of the anticodon loop in tRNA^Lys^, tRNA^Glu^, and tRNA^Gln^. Sulfur from cysteine is first transferred to IscS, then to TusA, and subsequently to the TusDCB complex, which delivers it to TusE and then to MnmA. Finally, MnmA transfers the sulfur atom to the uridine base at position 34 (10).

Sulfur modification of tRNAs, including tRNA^Lys^, tRNA^Glu^, and tRNA^Gln^, is thought to play a critical role in post-transcriptional regulation of gene expression by enhancing translation accuracy and efficiency (11). In mammalian cells, this modification is known to regulate various physiological functions. For example, loss of tRNA sulfur modification can cause mitochondrial diseases, whereas excessive modification has been implicated in tumorigenesis (12–14). In contrast, relatively few studies have examined the role of tRNA sulfur modification in regulating physiological functions in prokaryotes, including pathogenic bacteria.

We recently reported that TusDCB-mediated sulfur modification of tRNA^Lys^, tRNA^Glu^, and tRNA^Gln^ plays an important role in the pathogenicity of *E. coli* during urinary tract infection. Deletion of *tusDCB* impaired bacterial colonization and fitness in the bladder and kidneys of infected mice (15). Because sulfur modification of these tRNAs contributes to translation accuracy and efficiency, deletion of *tusDCB* is expected to cause global alterations in protein translation rather than affecting a single virulence factor. Thus, TusDCB-mediated tRNA modification may broadly influence the expression of multiple virulence-associated proteins across different *E. coli* pathotypes. Notably, some *E. coli* strains exhibit pathogenicity in the gastrointestinal tract, causing diarrhea and other enteric symptoms. To test this hypothesis, we focused on EHEC, a highly virulent intestinal pathogen. We constructed a *tusDCB* mutant and investigated the relationship between intestinal pathogenicity and TusDCB-mediated sulfur modification. Our experiments demonstrated that TusDCB is essential for optimal intestinal infection by supporting the expression of T3SS proteins, T3SS-dependent virulence, and bacterial fitness.

## MATERIALS AND METHODS

### Bacterial strains and growth conditions

Bacteria (EHEC O157:H7 Sakai [RIMD 0509952], *Citrobacter rodentium* DBS100 [ATCC51459], and its derivatives) were grown in Luria–Bertani (LB) broth (0.5% Yeast extract, 1% Tryptone, and 1% NaCl) or HEPES-free/high glucose (4,500 mg/L) Dulbecco’s modified Eagle medium (DMEM buffered with sodium bicarbonate) at 37°C with aeration by shaking. Cell growth was monitored by measuring the optical density at 600 nm (OD_600_). When required, antibiotics were added to the growth medium at the following concentrations: chloramphenicol (45 µg/mL) and kanamycin (50 µg/mL).

### Construction of the *tusDCB* deletion mutant

An in-frame deletion mutant of *tusDCB* was constructed using a homologous recombination method, following a previously described protocol (16). Primer pairs FW1/RV1 and FW2/RV2 (listed in Table 1) were used to amplify the upstream and downstream flanking regions of the *tusDCB* locus in EHEC and *C. rodentium*. The upstream fragment comprised 450 bp plus the first two codons of *tusD*, whereas the downstream fragment contained the last two codons of *tusB*, the stop codon, and 450 bp of downstream sequence. These DNA fragments were cloned into the temperature-sensitive vector pKO3 (17) using an In-Fusion cloning kit (Takara Bio, Shiga, Japan). The resulting plasmid was introduced into EHEC and *C. rodentium* strains. Sucrose-resistant, chloramphenicol-sensitive colonies were selected at 30°C to confirm allelic exchange.

**TABLE 1:** Primers used for mutant and plasmid constructions

Primer	DNA sequence (5′–3′)	Use
tusDCB-EHEC-delta-FW1	tcgcggccgcggaccggatcggttcgccaattactgacc	*EHEC tusDCB* mutant construction
tusDCB-EHEC-delta-RV1	cccgccatcacattacttatcttgcccctgg	*EHEC tusDCB* mutant construction
tusDCB-EHEC-delta-FW2	ataagtaatgtgatggcgggatcgttgtatatttc	*EHEC tusDCB* mutant construction
tusDCB-EHEC-delta-RV2	cattcgccattcccggtcgattaagccttaggacgcttcacg	*EHEC tusDCB* mutant construction
tusDCB-CR-delta-FW1	tcgcggccgcggaccggatcggttcgccgattaccgacc	*C. rodentium tusDCB* mutant construction
tusDCB-CR-delta-RV1	gccgtcaccaacgcattacttatcttgcccctgg	*C. rodentium tusDCB* mutant construction
tusDCB-CR-delta-FW2	agtaatgcgttggtgacggcgcgatcgttg	*C. rodentium tusDCB* mutant construction
tusDCB-CR-delta-RV2	attcgccattctccggtcgattaagccttaggacgcttcacg	*C. rodentium tusDCB* mutant construction
dauR-F	gcgggatccgcgaaagccgcagactctgc	pTH18kr-dauRtusDCB construction
pTHtusB-R	gcgaagctttcaccaggccatctggctgg	pTH18kr-dauRtusDCB construction

### Complementation of *tusDCB*

For genetic complementation, the *tusDCB* operon was amplified together with 196 bp upstream of *dauR* and cloned into the low-copy-number plasmid pTH18kr (18) via BamHI and HindIII restriction sites, yielding pTH18kr-dauRtusDCB. PCR amplification was performed with primers dauR-F and pTHtusB-R. All constructs were confirmed by DNA sequencing.

### Shiga toxin assay

Shiga toxin 1 (Stx1) and Shiga toxin 2 (Stx2) levels were quantified using latex agglutination kits (Denka Seiken Co., Ltd., Tokyo, Japan) with minor modifications to the manufacturer’s protocol. EHEC strains were grown in DMEM at 37°C with shaking to the early stationary phase. Culture supernatants were serially diluted in 96-well round-bottom plates containing phosphate-buffered saline (PBS). Latex beads coated with anti-Stx1 or anti-Stx2 antibodies were added, and plates were incubated for 16 h at 4°C. Titers were determined as the reciprocal of the highest dilution yielding visible agglutination.

### Western blotting

Bacterial cultures were grown in DMEM to the early stationary phase, harvested by centrifugation, and supernatants were filtered. Secreted proteins were precipitated from the supernatants with 10% trichloroacetic acid (TCA) and dissolved in Laemmli sample buffer. Bovine serum albumin (BSA) was used as a loading control and was added to the secreted protein samples prior to the precipitation with TCA. Intracellular proteins were extracted in 50 mM phosphate buffer containing 8 M urea and lysed by sonication. Protein samples (6 µg) were separated on 12.5% SDS–polyacrylamide gels and electroblotted onto PVDF membranes. EspB, EspA, and DnaK were detected using anti-EspB and anti-EspA antisera (19) and anti-DnaK antibody (Abcam, Cambridge, UK), respectively, followed by horseradish peroxidase–conjugated secondary antibodies (Sigma-Aldrich, St. Louis, MO, USA) and visualized using Chemi-Lumi One (Nacalai Tesque, Kyoto, Japan). Images were acquired using an LAS-4000 Luminescent Image Analyzer (GE Healthcare Japan, Tokyo, Japan) and processed with ImageQuant LAS 4000 software.

### RNA extraction and quantitative real-time PCR

Bacteria were grown in DMEM to mid-logarithmic phase. Total RNA was extracted using the Monarch Total RNA Miniprep Kit (New England Biolabs, Ipswich, MA, USA), and cDNA synthesis was carried out using the ReverTra Ace qPCR RT Master Mix (Toyobo Co., Ltd., Osaka, Japan). Genomic DNA contamination was eliminated by on-column DNase I digestion followed by treatment with gDNA remover supplied in the RT kit. Quantitative PCR reactions contained 2 ng of cDNA and 160 nM of each primer in Thunderbird Next SYBR qPCR Mix (Toyobo Co., Ltd.). *rrsA* and *rpoD* served as internal reference genes. Primer sequences are listed in Table 2.

**TABLE 2:** Primers used for quantitative PCR in this study

Primer	DNA sequence (5′–3′)	Primer	DNA sequence (5′–3′)
rrsA-qPCR-F	cggtgaagcatgtgttaa	rrsA-qPCR-R	gaaaacttccatggatcaaag
rpoD-qPCR-F	gaagccgtgctcgaaaa	rpoD-qPCR-R	gggcgcgtatgcactttct
ler-qPCR-F	gcaccagtctccccttct	ler-qPCR-R	tccctcgcccgaactc
espA-qPCR-F	ccgttctacgtttatccgtt	espA-qPCR-R	tgatttaacccctggttactg
tir-qPCR-F	ttttcgcgcctgagctatt	tir-qPCR-R	gctaaagacgcaaggcgaaga
escJ-qPCR-F	aaaagaactaatcagatgcaagca	escJ-qPCR-R	tccactttttacactttttgga
escV-qPCR-F	cgctctccgtacagaaa	escV-qPCR-R	ttatctatcgatgctactttg
stx1-qPCR-F	tcgcgagttcgaaaagta	stx1-qPCR-R	ttccatctgcacggacacat
stx2-qPCR-F	ttcgccgtgaaatgaa	stx2-qPCR-R	caggccgtctgccagttac

### Hemolysis assay

T3SS-dependent hemolytic activity was determined as described previously (20). EHEC strains were grown statically in DMEM overnight at 37°C in 5% CO_2_. Human red blood cells (RBCs) were washed and resuspended to 3 × 10^9^ cells/mL in DMEM; bacteria were resuspended to 7 × 10^9^ cells/mL. Fifty microliters of each suspension were mixed in 96-well flat-bottom plates. RBCs in DMEM without bacteria served as negative controls, whereas RBCs lysed with distilled water served as positive controls. After 3 h of incubation at 37°C in 5% CO_2_, 100 µL of DMEM was added to each well, plates were centrifuged, and 100 µL of supernatant was transferred to ELISA plates. Hemolysis was quantified by measuring absorbance at 450 nm. Percent hemolysis was calculated as: {(sample) − (negative control)}/{(positive control) − (negative control)} × 100.

### *C. rodentium* infection model in mice

*C. rodentium* DBS100 and its *tusDCB* mutant were cultured aerobically overnight in LB medium at 37°C. Cells were harvested, resuspended in fresh LB to 1 × 10^9^ colony-forming units (CFU)/mL, and 200 µL of suspension (2 × 10^8^ CFU) was orally administered to 4-week-old female C3H/HeJ mice (Japan SLC, Inc., Shizuoka, Japan) (*n* = 5 per group). Control mice received 200 µL of sterile LB broth and/or a culture suspension of the non-pathogenic *E. coli* K-12 strain MG1655 (ATCC700926). Mice were monitored daily for 30 days to record survival, and body weight. To assess intestinal pathology, mice were euthanized 9 days post-infection, and colons were aseptically excised.

### Bile salt susceptibility assays

Bacterial susceptibility to bile salts was determined by measuring the minimum inhibitory concentrations (MICs) of sodium deoxycholate and sodium cholate, following established protocols (21). Five microliters of 100-fold-diluted overnight cultures (~50,000 cells) were inoculated onto an LB agar plate containing sodium deoxycholate or sodium cholate and incubated for 18 h at 37°C. The MICs were determined as the lowest concentration at which growth was inhibited.

### Acid survival assays

Bacteria were grown to late logarithmic phase in DMEM at 37°C, then replaced in acidified LB (pH 3.5, 3.0, or 2.5; adjusted with HCl) and incubated for 1 h. Survival was expressed as the percentage of CFU counts after incubation in acidified medium relative to CFU counts prior to acid exposure.

### Mass spectrometry analysis

EHEC strains were cultured in DMEM at 37°C with shaking to early stationary phase, harvested, resuspended in PTS buffer (22), and lysed by sonication for proteomic analysis. Equal amounts of the protein (10 µg) were digested by trypsin and LysC, and analyzed by LC-MS/MS. In detail, the proteins were incubated with 10 mM dithiothreitol (Fujifilm Wako Pure Chemical Industries, Osaka, Japan) in 50 mM ammonium bicarbonate solution for 30 min at room temperature, then incubated with 50 mM iodoacetamide (Fujifilm Wako Pure Chemical Industries) for 30 min at room temperature in the dark. The reaction was terminated by adding four volumes of 50 mM ammonium bicarbonate solution. The proteins were digested with 0.5 µg trypsin (Promega Corp., Madison, WI, USA) and 0.1 µg LysC (Fujifilm Wako Pure Chemical Industries) overnight at 37°C. Sodium deoxycholate and sodium lauroyl sarcosinate in the PTS buffer were removed by ethyl acetate extraction after the acidification of the samples by formic acid. The digested peptides were desalted with C18 stage GL-tips (GL Sciences Inc., Tokyo, Japan), dried up, then resuspended in water containing 0.1% formic acid, and applied to the LC-MS/MS analysis with an Eksigent Ekspert NanoLC 425 system coupled to a TripleTOF 6600 mass spectrometer (Sciex). The peptide mixture was separated by an ODS column (Eksigent ChromXP-C18-CL, 3 μm, 120 Å, 0.075 mm I.D. × 150 mm L, Sciex) with 2–30% acetonitrile gradient containing 0.1% formic acid for 60 min. Protein identification was performed with the Paragon algorithm search engine using a ProteinPilot software (version 5.0.2, Sciex). For the quantitative analysis of the proteins, sequential window acquisition of all theoretical mass spectra (SWATH-MS) proteomics was performed by using the PeakView software (version 2.2, Sciex) according to the manufacturer’s instructions.

### Indole assay

Indole production was quantified as previously described (19). Bacteria were grown in DMEM supplemented with 2.5 mM tryptophan for 24 h, and indole was extracted from culture supernatants with ethyl acetate. Kovac’s reagent (0.7 mL) was added to 0.02 mL of the ethyl acetate fraction, and absorbance at 540 nm was measured. Indole concentrations were calculated from a standard curve generated with commercial indole (Fujifilm Wako Pure Chemical Industries).

## RESULTS

### Effect of *tusDCB* deletion on Shiga toxin and EspB secretion

**Fig 1:**
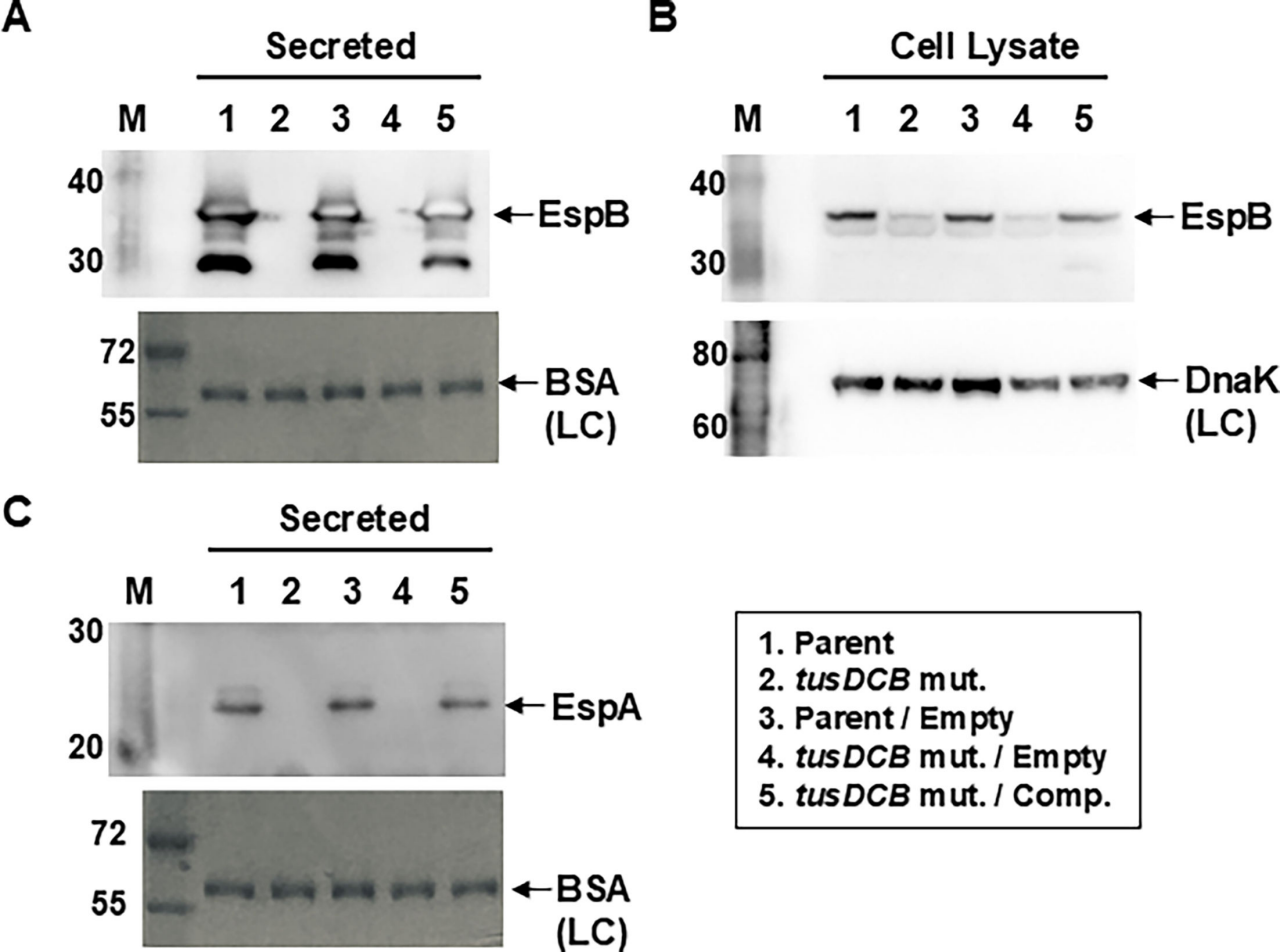
Detection of (**A and B**) EspB and (**C**) EspA in culture supernatants and whole-cell extracts of the parent strain and *tusDCB* mutant, or the parent and *tusDCB* mutant carrying pTH18kr (empty vector) or pTH18kr-dauRtusDCB (complementation plasmid). DnaK and BSA were used for loading controls (LCs). Proteins were separated by SDS–PAGE, and EspB, EspA and DnaK were visualized by Western blotting using anti-EspB and anti-EspA antisera and anti-DnaK antibody, respectively. BSA was visualized by Coomassie brilliant blue (CBB) stain. Molecular mass standards (kDa) are indicated on the left.

To investigate the role of tRNA sulfur modification in EHEC pathogenicity, we constructed a *tusDCB* deletion mutant in the EHEC Sakai strain background. EHEC virulence is primarily mediated by two protein sets: Shiga toxins (Stx1 and Stx2) and type III secretion system (T3SS) proteins, such as the translocator EspB (2). We compared the levels of Stx1, Stx2, and EspB secreted by the parent and *tusDCB* mutant strains. Stx1 and Stx2 levels were quantified by latex agglutination assays, and EspB levels were analyzed by SDS–PAGE followed by Western blotting. The *tusDCB* mutant produced only approximately twofold lower titers of Stx1 than the parent strain, while Stx2 titers showed no significant difference (Table 3). In contrast, EspB levels in the secretion fraction were markedly reduced in the *tusDCB* mutant. Complementation of the mutant with the *tusDCB*-expressing plasmid pTH18k-dauRtusDCB restored EspB levels to those of the parent strain (Fig. 1A). Intracellular EspB levels were also reduced in the mutant, although the reduction was less pronounced than that observed in the secretion fraction (Fig. 1B). To determine whether this effect was specific to EspB or reflected a broader defect in T3SS secretion, we also examined the level of another secreted T3SS protein, EspA. Similar to EspB, the level of EspA in the secretion fraction was markedly reduced in the *tusDCB* mutant and was restored to the parental level by genetic complementation (Fig. 1C).

**TABLE 3:** Shiga toxin titers in the parent and *tusDCB* mutant EHEC strains

	Shiga toxin titers	
Strains	Stx1	Stx2
Parent	128	32
tusDCB mut.	64	32

**Fig 2:**
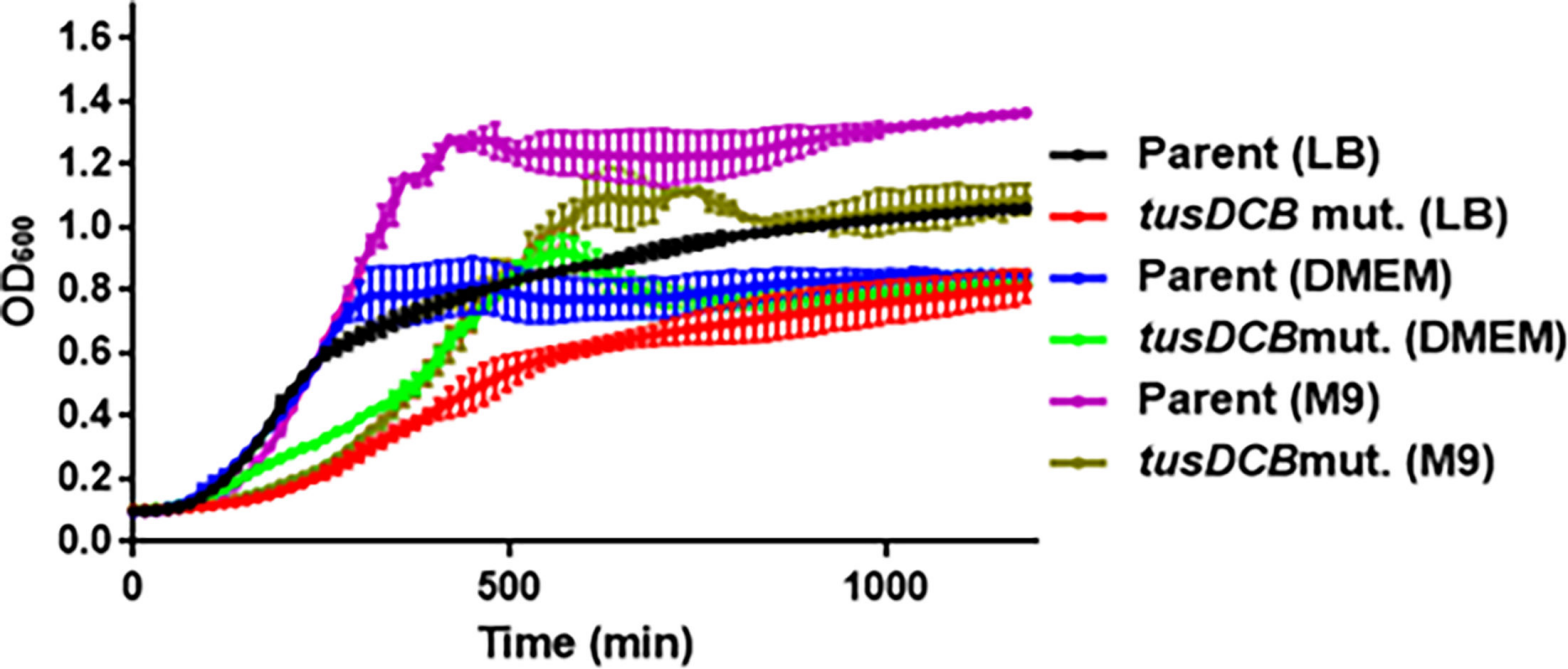
Growth of the parent strain and *tusDCB* mutant cultured in LB medium, DMEM, M9 medium containing 0.5% casamino acids and 0.5% glucose. Bacterial growth was monitored by measuring OD_600_. Data are means of two biological replicates; error bars indicate the ranges. Similar results were obtained in two independent experiments.

To examine whether the *tusDCB* mutation affects bacterial growth, we compared the growth curves of the parent and *tusDCB* mutant strains in LB, M9, and DMEM (Fig. 2). In LB and M9 media, the *tusDCB* mutant showed a clear growth delay compared with the parental strain. In DMEM, the mutant also exhibited a slight delay in the early growth phase, although the difference was smaller than that observed in LB or M9. Importantly, both strains reached comparable OD_600_ and CFU levels at the stationary phase in DMEM. Because the cultures used for Western blot analysis were harvested at the stationary phase, the reduced EspB and EspA levels observed in the *tusDCB* mutant are unlikely to be explained solely by differences in bacterial growth.

### Transcriptional regulation of T3SS genes by *tusDCB*

**Fig 3:**
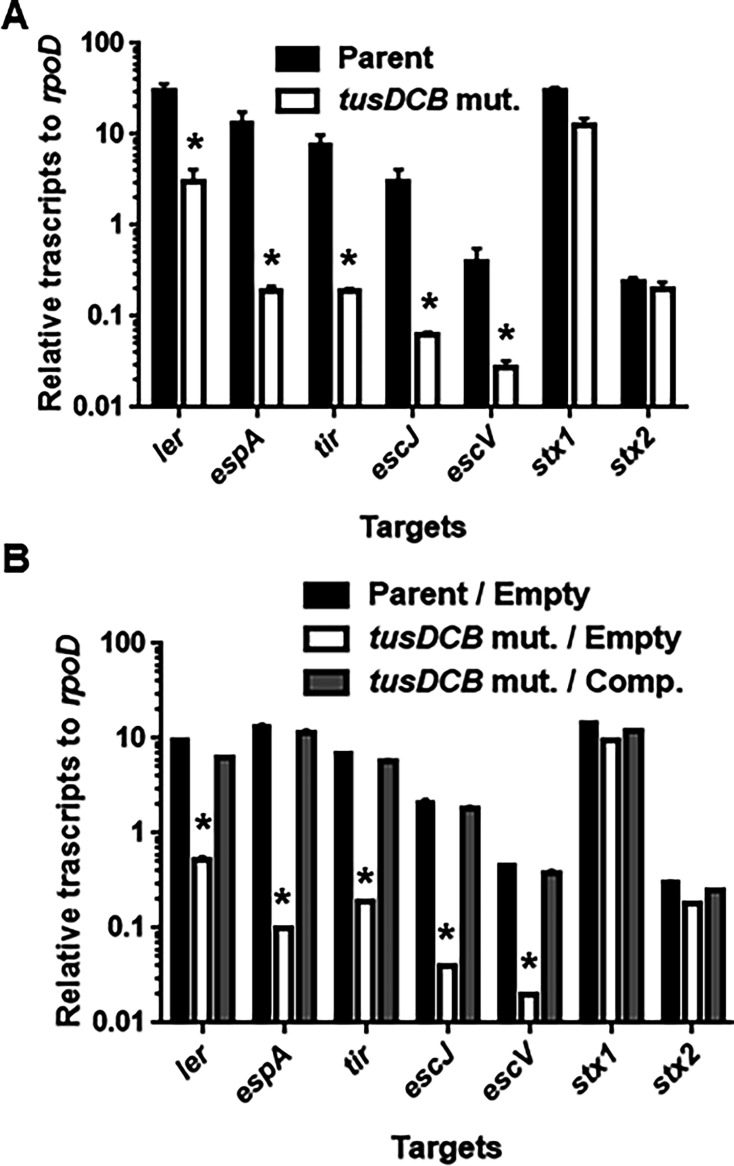
Relative transcript levels of LEE operon genes and *stx* (*stx1* and *stx2*) in (**A**) the parent strain and the *tusDCB* mutant (*tusDCB* mut.), or (**B**) the parent and *tusDCB* mutant carrying pTH18kr (empty vector) or pTH18kr-dauRtusDCB (complementation plasmid), normalized to *rpoD*. Data represent means of four biological replicates; error bars indicate standard deviations. * indicate statistical significance (*P* < 0.05) versus the parental strain (unpaired *t*-test).

We next examined whether *tusDCB* deletion affected the transcription of genes encoding T3SS proteins. The LEE pathogenicity island, consisting of five major operons (LEE1–LEE5), encodes T3SS structural components, translocators, and effectors, including EspB. The *espB* gene resides in the LEE4 operon, downstream of *espA* (6). We quantified transcript levels of representative genes from each operon (*ler*, *espA*, *tir*, *escJ*, and *escV*), including the master regulator Ler, using quantitative PCR. Transcript levels of all these genes were significantly lower in the *tusDCB* mutant than in the parent strain (Fig. 3A). In contrast, the transcript levels of *stx1* and *stx2* were not significantly affected by *tusDCB* deletion. Importantly, the reduced transcript levels of the T3SS genes in the *tusDCB* mutant were restored to levels comparable to those of the parent strain by complementation with the *tusDCB*-expressing plasmid (Fig. 3B). These results indicate that *tusDCB* contributes to the regulation of T3SS gene expression, thereby influencing EspB production and secretion. These findings are consistent with the reduced secretion of T3SS proteins observed in the *tusDCB* mutant.

### T3SS-dependent hemolysis and *in vivo* virulence

**Fig 4:**
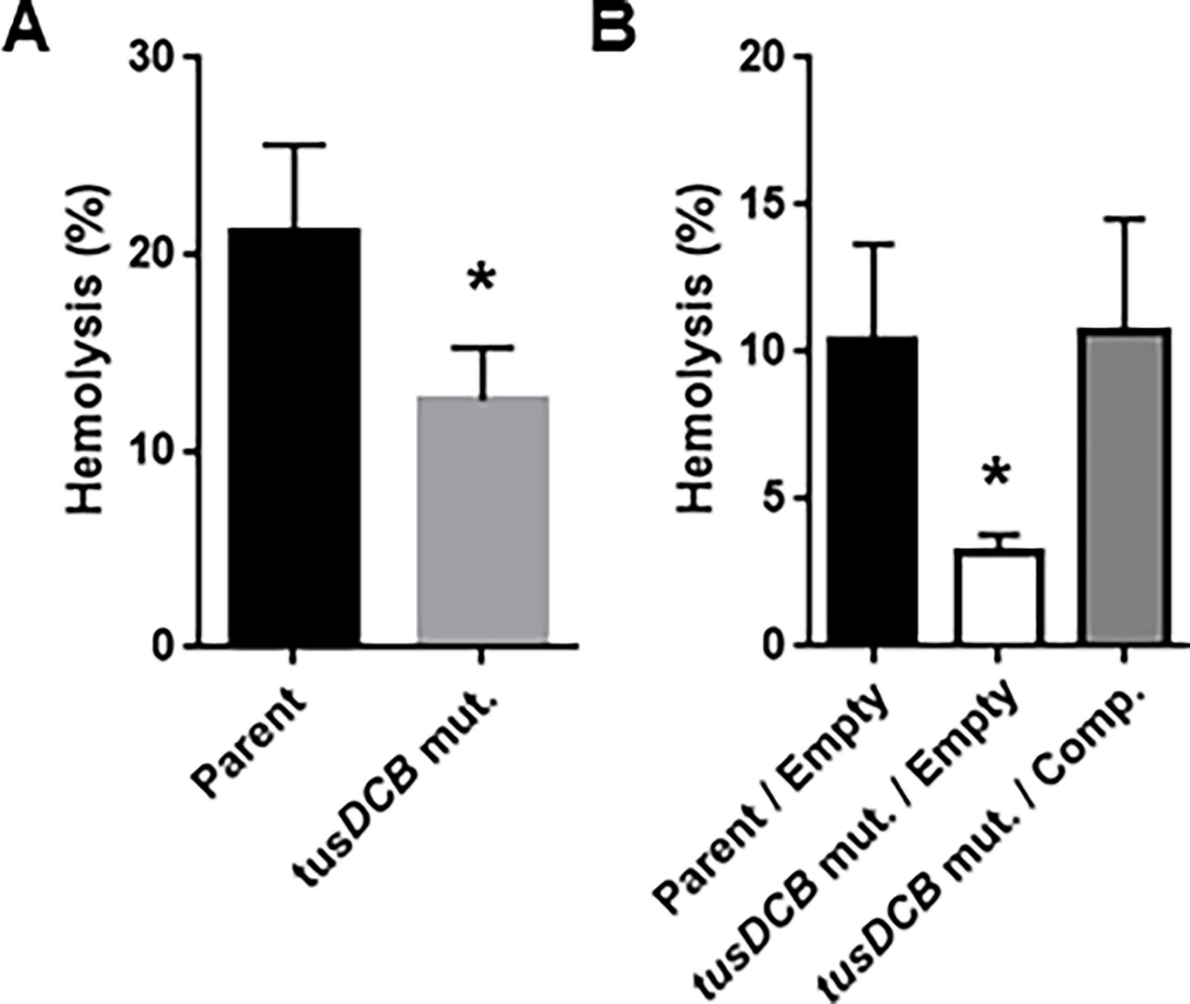
T3SS-dependent hemolysis of human red blood cells (RBCs) by (**A**) the parent strain and the *tusDCB* mutant, or (**B**) the parent and *tusDCB* mutant carrying pTH18kr (empty vector) or pTH18kr-dauRtusDCB (complementation plasmid). Hemolysis (%) is expressed as the proportion of lysed RBCs (see Materials and Methods). Data represent means of three biological replicates; error bars indicate standard deviations. * indicate statistical significance *(P* < 0.05) versus parent strain (unpaired *t*-test).

To assess whether reduced T3SS expression in the *tusDCB* mutant affected T3SS-dependent virulence, we measured hemolytic activity toward human red blood cells. The *tusDCB* mutant exhibited approximately two-fold lower hemolytic activity than the parent strain (Fig. 4A). Complementation of the mutant with a *tusDCB*-expressing plasmid restored hemolytic activity to a level comparable to that of the parent strain (Fig. 4B).

**Fig 5:**
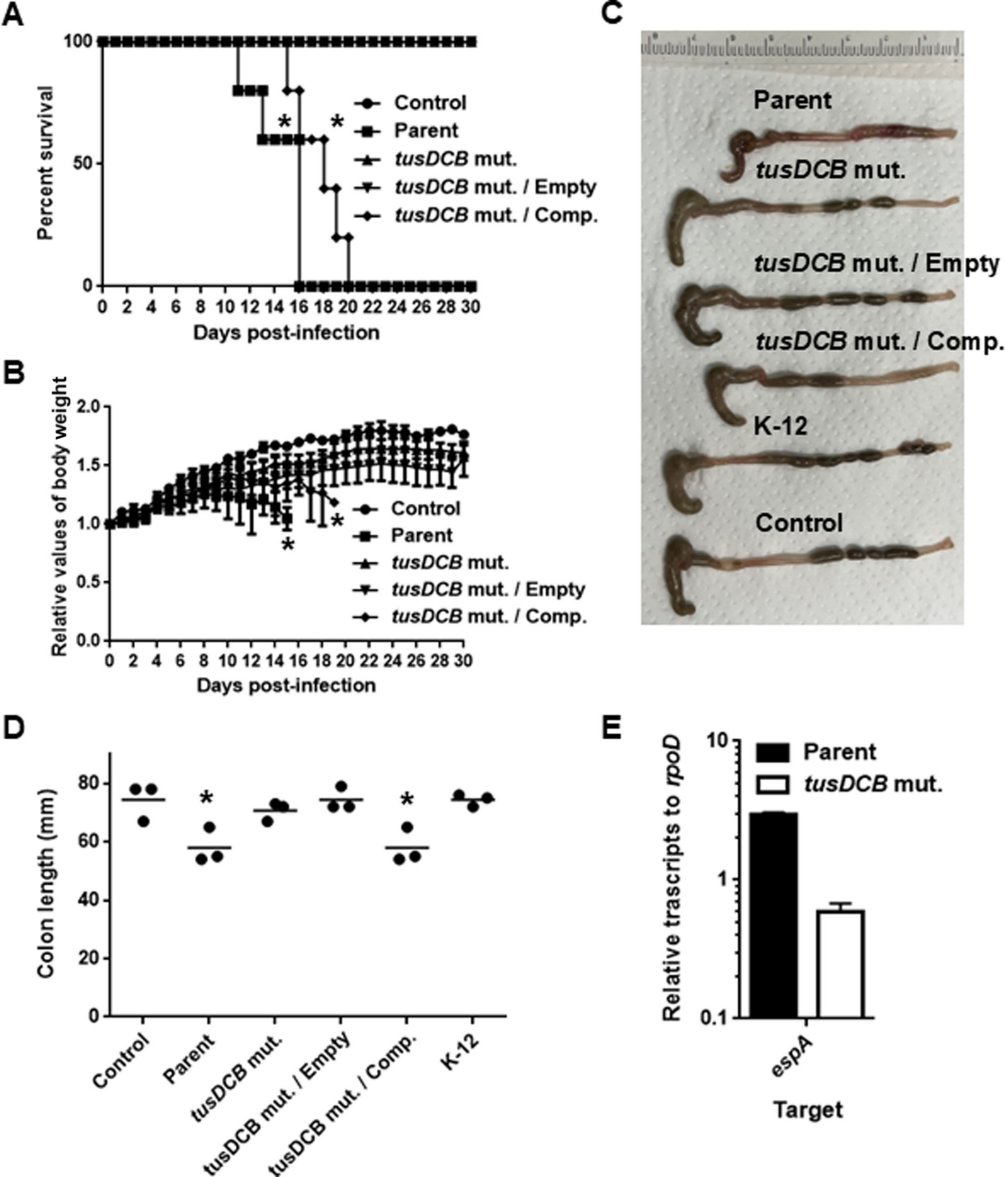
Virulence of parent and *tusDCB* mutant *C. rodentium* strains in C3H/HeJ mice. (**A**) Survival curves, and (**B**) body weight change in mice infected with the parent strain, the *tusDCB* mutant, the *tusDCB* mutant carrying pTH18kr (empty vector) or pTH18kr-dauRtusDCB (complementation plasmid), or uninfected controls. Lines denote means; error bars indicate standard deviations. (**C and D**) Colon length in mice infected with the parent strain, the *tusDCB* mutant, the *tusDCB* mutant carrying pTH18kr (empty vector) or pTH18kr-dauRtusDCB (complementation plasmid), or non-pathogenic *E. coli* K-12, and uninfected controls at 9 days post-infection. Each point represents an individual mouse; horizontal bars indicate geometric means. (**E**) Relative transcript levels of *espA* in the parent and *tusDCB* mutant *C. rodentium* strains, normalized to *rpoD*. Data represent means of four biological replicates; error bars indicate standard deviations. * indicate statistical significance *(P* < 0.05) versus the parent strain (Gehan–Breslow–Wilcoxon test for mouse survival experiments and unpaired *t*-test for body weight and colon length measurements and qPCR analyses).

We further examined virulence *in vivo* using a murine infection model with the alternative intestinal pathogen *C. rodentium*, which expresses T3SS proteins orthologous to those of EHEC and induces severe diarrhea in mice (23). A *tusDCB* deletion mutant was constructed in the *C. rodentium* DBS100 background and orally inoculated into C3H/HeJ mice. All mice infected with the parent strain died within 16 days, whereas all mice infected with the *tusDCB* mutant survived for at least 30 days, similar to the uninfected control group (Fig. 5A). Body weight was monitored over time. Parent strain-infected mice exhibited rapid weight loss beginning on day 12 post-infection (Fig. 5B). In contrast, mice infected with the *tusDCB* mutant showed only mild transient weight loss followed by recovery (Fig. 5B). Complementation of the *tusDCB* mutant restored virulence, resulting in weight loss kinetics comparable to those observed in mice infected with the parent strain (Fig. 5A and B). Gross examination of colons at day nine post-infection revealed severe inflammation, edema, and focal hemorrhage in parent strain-infected mice, accompanied by marked colon shortening (Fig. 5C and D). These pathological changes were absent in mice infected with the *tusDCB* mutant or with the non-pathogenic *E. coli* K-12 strain (Fig. 5C). Colon length measurements confirmed significant shortening in parent strain-infected mice but not in *tusDCB* mutant-infected mice, whereas complementation restored the colon-shortening phenotype (Fig. 5D).

Because the *in vivo* infection experiments were performed using *Citrobacter rodentium*, we next examined whether *tusDCB* deletion also affected T3SS gene expression in this organism. Due to the lack of cross-reactivity of our anti-EspA and anti-EspB antisera with *C. rodentium*, we assessed transcription of the *espA* gene by quantitative PCR. The *espA* gene is located in the same operon as *espB* and encodes a key component of the T3SS filament. The *tusDCB* mutant exhibited significantly lower *espA* transcript levels than the parent strain, indicating that *tusDCB* also contributes to T3SS gene expression in *C. rodentium* (Fig. 5E). Together, these results demonstrate that *tusDCB* contributes to T3SS-dependent intestinal virulence.

### Sensitivity to bile salts, acid, and oxidative stress

Because enteric pathogens encounter bile salts in the intestine, we compared the susceptibility of the parent and *tusDCB* mutant strains to sodium deoxycholate and sodium cholate. Minimum inhibitory concentrations (MICs) were similar for both strains (Table 4), indicating that reduced virulence of the *tusDCB* mutant is not attributable to increased bile salt sensitivity.

**TABLE 4:** Bile salt MICs for EHEC and *C. rodentium*, and its *tusDCB* mutants

	MICs (mg/L)	
Strain	Sodium deoxycholate	Sodium cholate
Parent (EHEC)	>51,200	>51,200
tusDCB mut. (EHEC)	>51,200	>51,200
Parent (*C. rodentium*)	3,200	51,200
tusDCB mut. (*C. rodentium*)	3,200	51,200

**Fig 6:**
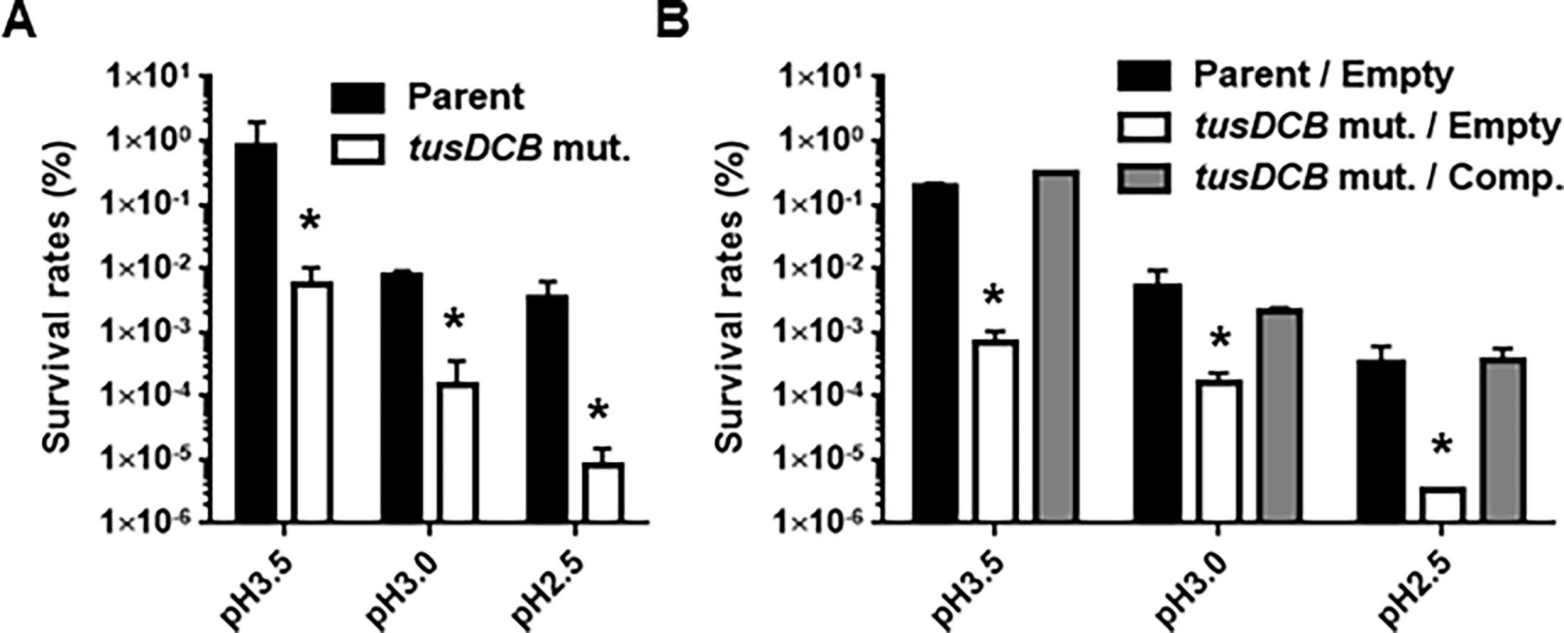
Susceptibility of (**A**) EHEC parent and *tusDCB* mutant, or (**B**) the parent and *tusDCB* mutant carrying pTH18kr (empty vector) or pTH18kr-dauRtusDCB (complementation plasmid) strains to acid stress. Survival (%) was calculated as CFU counts after incubation in acidified LB (pH 3.5, 3.0, or 2.5) relative to CFU counts in non-acidified LB. Data represent means of three biological replicates; error bars indicate standard deviations. * indicate statistical significance (*P* < 0.05) versus the parent strain (unpaired *t*-test).

**Fig 7:**
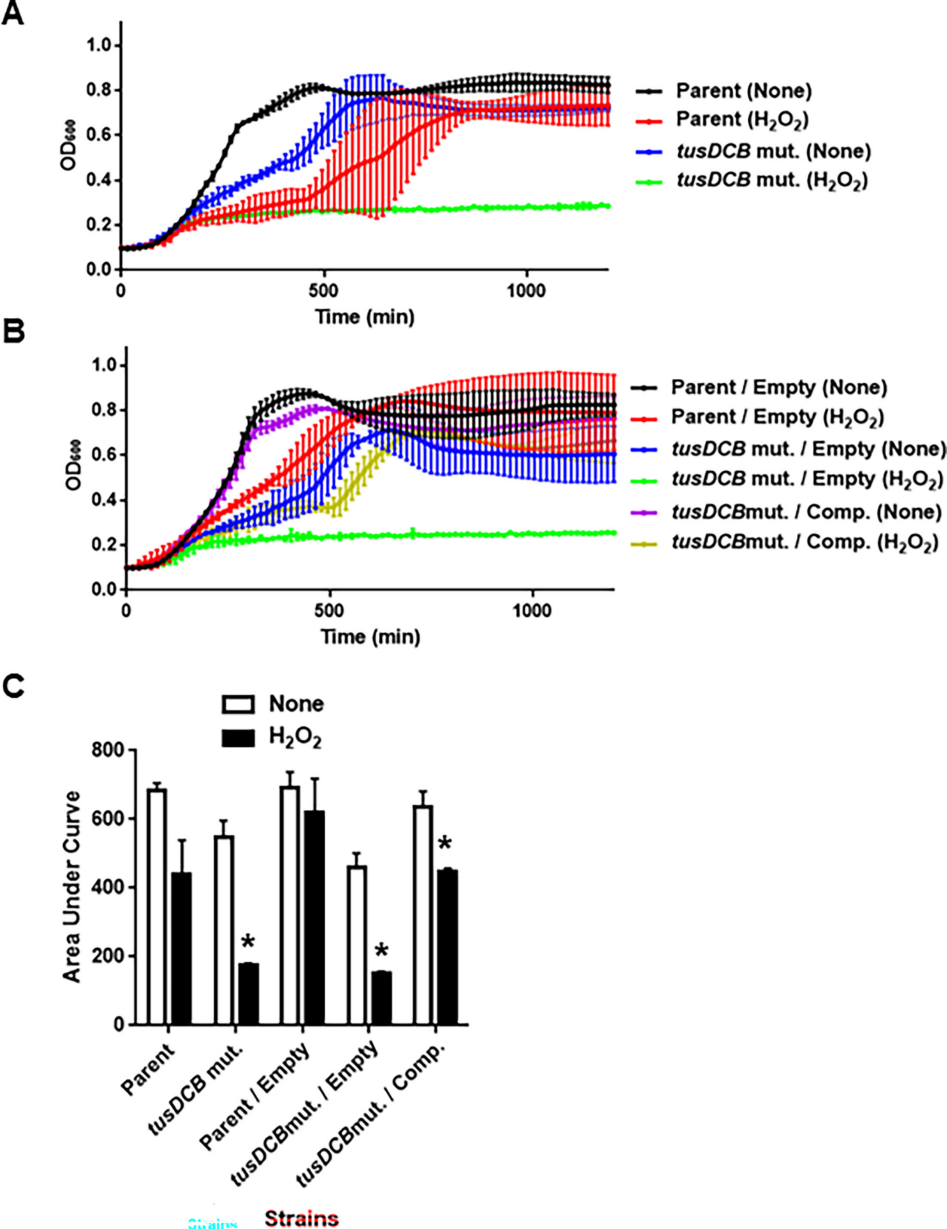
Susceptibility of (**A**) EHEC parent and *tusDCB* mutant, or (**B**) the parent and *tusDCB* mutant carrying pTH18kr (empty vector) or pTH18kr-dauRtusDCB (complementation plasmid) strains to oxidative stress. Growth in DMEM with or without hydrogen peroxide (0.78mM) was monitored by OD_600_. (**C**) Quantification of growth by area under the curve (AUC) analysis of the growth curves shown in panels **A **and **B**. Data are from two biological replicates; error bars indicate ranges. Similar results were obtained in two independent experiments. * indicate statistical significance *(P* < 0.05) versus control culture (None) (unpaired *t*-test).

To evaluate acid tolerance, we exposed the parent strain and the *tusDCB* mutant to LB medium adjusted to pH 2.5, 3.0, or 3.5 for 1 h. Survival of the *tusDCB* mutant was more than 100-fold lower than that of the parent strain under acidic conditions, demonstrating a marked defect in acid resistance (Fig. 6A). Complementation of the mutant with a *tusDCB*-expressing plasmid restored acid tolerance to a level comparable to that of the parent strain (Fig. 6B). We also tested sensitivity to oxidative stress using hydrogen peroxide. At 0.78 mM H_2_O_2_, the parent strain showed only a slight growth delay, whereas the *tusDCB* mutant exhibited a marked growth defect, consistent with the area under the curve analysis (Fig. 7A and C). This oxidative stress sensitivity was also rescued by complementation with *tusDCB* (Fig. 7B and C). These findings suggest that TusDCB contributes to intrinsic resistance to both acid and oxidative stress conditions that bacteria encounter during passage through and colonization of the gastrointestinal tract.

### Proteomic analysis and indole production

To identify proteins whose expression depends on TusDCB, we performed mass spectrometry-based proteomic analysis comparing the parent and *tusDCB* mutant EHEC strains. Of approximately 5,350 predicted proteins in the EHEC O157:H7 Sakai genome (24), 1,996 were detected (~38%) (Fig. 10). Most of these proteins showed reduced expression levels in the *tusDCB* mutant compared to the parent strain, suggesting that loss of TusDCB function may cause a general decrease or delay in protein translation. Twenty-one proteins showed more than twofold lower abundance in the *tusDCB* mutant compared with the parent strain (*P* < 0.05) (Table 5). Among the proteins significantly decreased in the *tusDCB* mutant, TnaA, the enzyme responsible for indole production, showed one of the most pronounced reductions.

**TABLE 5:** Proteins with significantly reduced expression (>2-fold) in the *tusDCB* mutant compared with the parent EHEC strain (*P* < 0.05)

Protein ID	Proteins	Ratio (Parent/Mut)^a^
ECs_2711	ZinT zinc and cadmium binding protein	4.80
ECs_3417	GlyA serine hydroxymethyltransferase	4.72
ECs_3397	IscR transcriptional regulator	3.79
ECs_4792	GlnA glutamine synthetase	3.27
ECs_0327	YkgM 50S ribosomal protein L31 type B	3.22
ECs_3460	RaiA translation inhibitor protein	3.06
ECs_5196	YtfL inner membrane protein	3.02
ECs_4365	PitA phosphate transporter	2.88
ECs_2558	YebG hypothetical protein	2.74
ECs_3974	UxaC uronate isomerase	2.72
ECs_4645	TnaA tryptophanase	2.57
ECs_3656	SdaC serine transporter	2.43
ECs_4660	PhoU negative regulator of PhoR/PhoB two-component regulator	2.25
ECs_0485	CyoB cytochrome o ubiquinol oxidase subunit I	2.25
ECs_4554	EspB T3SS translocator	2.24
ECs_4870	MetF 5,10-methylenetetrahydrofolate reductase	2.24
ECs_4790	GlnG nitrogen regulation protein NR(I)	2.18
ECs_1005	MukF chromosome condensin MukBEF kleisin-like subunit	2.15
ECs_3430	PdxJ pyridoxine 5′-phosphate synthase	2.13
ECs_0777	YbgF periplasmic TolA-binding protein	2.04
ECs_3432	Era GTP-binding protein Era	2.04

^a^ Ratio indicates the relative abundance of each protein in the parent strain compared with the *tusDCB* mutant.

**Fig 8:**
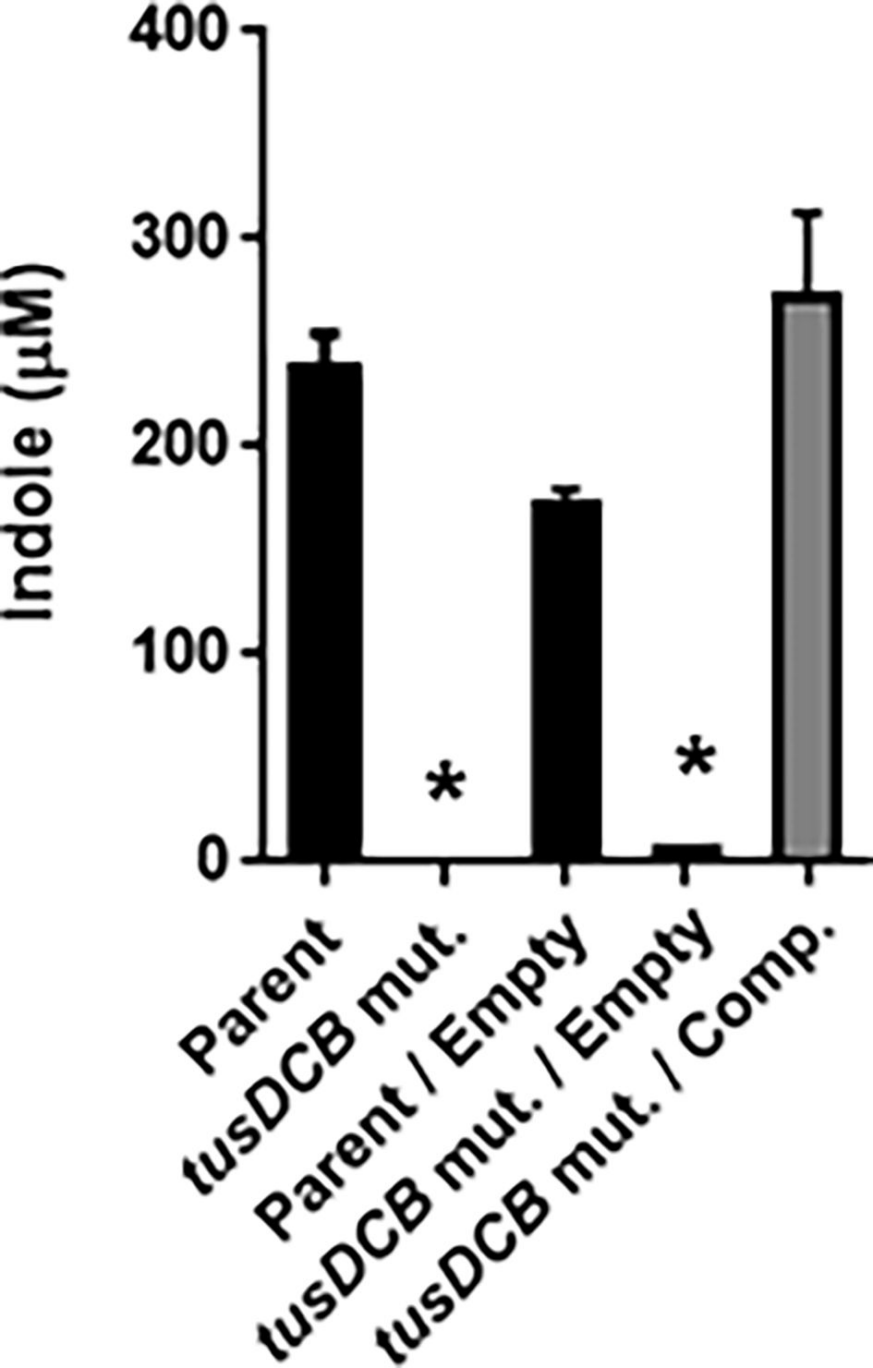
Indole production of EHEC parent and *tusDCB* mutant, or the parent and *tusDCB* mutant carrying pTH18kr (empty vector) or pTH18kr-dauRtusDCB (complementation plasmid) strains. indole concentrations in culture supernatants of strains grown in DMEM containing 2.5 mM tryptophan were measured. data represent means of three independent experiments; error bars indicate standard deviations. * indicate *(P* < 0.05) versus the parent strain (unpaired *t*-test).

**Fig 9:**
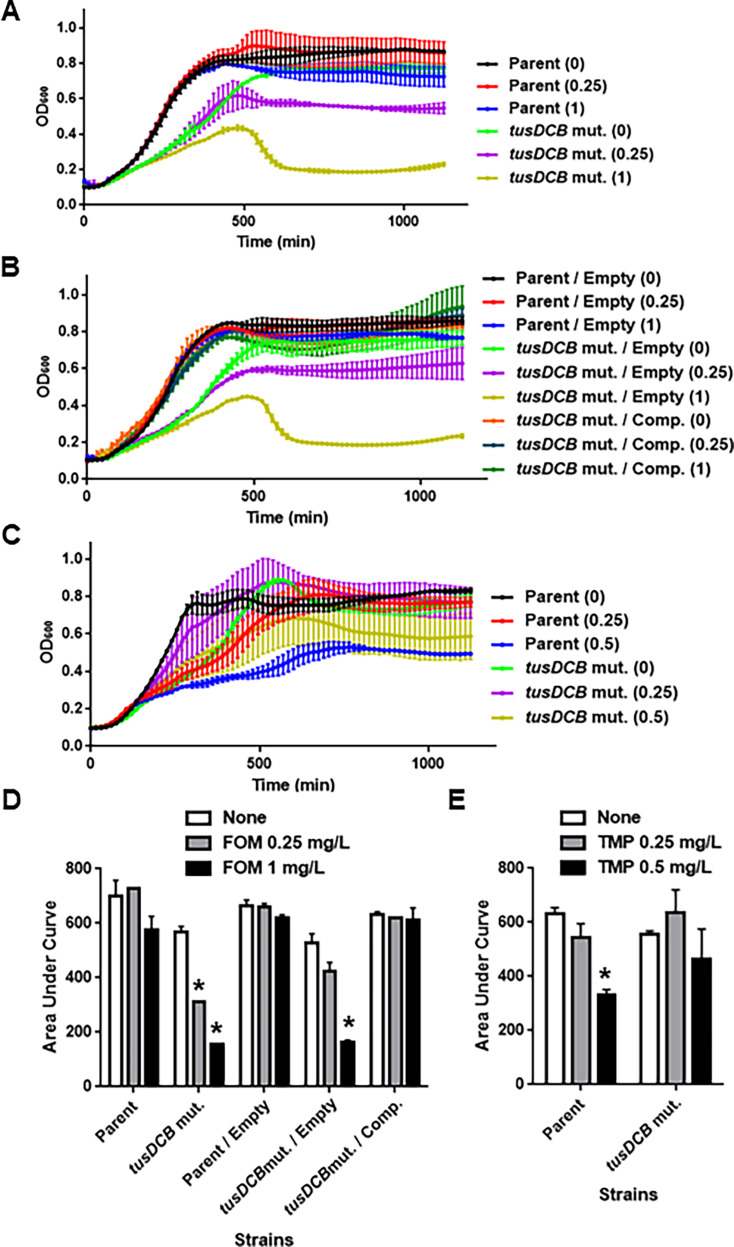
Susceptibility of EHEC parent and *tusDCB* mutant, or the parent and *tusDCB* mutant carrying pTH18kr (empty vector) or pTH18kr-dauRtusDCB (complementation plasmid) strains to (**A and B**) fosfomycin and (**C**) sulfamethoxazole/trimethoprim. Growth in DMEM with or without the indicated concentrations (mg/L) of fosfomycin or sulfamethoxazole/trimethoprim (5:1) was monitored by OD_600_. (**D and E**) Quantification of growth by area under the curve (AUC) analysis of the growth curves shown in panels A-C. Data are from two biological replicates; error bars indicate ranges. similar results were obtained in two independent experiments. * indicate *(P* < 0.05) versus control culture (None) (unpaired *t*-test).

TnaA catalyzes the conversion of tryptophan to indole, a signaling molecule that has been reported to enhance T3SS gene expression and confer resistance to certain antibiotics, including fosfomycin (25–27). Consistent with the loss of TnaA expression observed in the proteomic analysis, indole production by the *tusDCB* mutant grown in DMEM containing 2.5 mM tryptophan was below the detection limit, similar to that of a *tnaA* deletion mutant, whereas the parent strain produced approximately 300 µM indole (Fig. 8). Complementation of the mutant with a plasmid expressing *tusDCB* restored indole production to levels comparable to those of the parent strain (Fig. 8). Fosfomycin susceptibility assays further showed that the parent strain tolerated 1 mg/L fosfomycin with only mild growth inhibition, whereas the *tusDCB* mutant exhibited a marked growth defect under the same conditions (Fig. 9A and D). This phenotype was reversed by genetic complementation with the *tusDCB*-expressing plasmid (Fig. 9B and D). In contrast, susceptibility to sulfamethoxazole/trimethoprim, which is not known to be influenced by indole signaling, was similar between the parent and mutant strains (Fig. 9C and E). Because indole has been reported to enhance T3SS gene expression and fosfomycin resistance, we further tested whether exogenous indole could restore these phenotypes in the *tusDCB* mutant. However, supplementation with indole did not rescue the defects in T3SS expression or fosfomycin susceptibility (data not shown). These observations suggest that although TusDCB is required for indole production, the phenotypes observed in the *tusDCB* mutant cannot be explained solely by the loss of indole signaling and are likely influenced by additional effects of TusDCB-mediated tRNA sulfur modification on protein translation.

## DISCUSSION

Sulfur modification of tRNAs contributes to accurate codon recognition and optimal translation efficiency, and is conserved in both prokaryotes and eukaryotes (11, 28). However, the enzymes responsible for this modification differ between prokaryotes and eukaryotes. In *E. coli*, TusDCB plays a central role in this process. Homologs of TusDCB are found in some γ-proteobacteria and environmental bacteria such as *Allochromatium vinosum*, *Chlorobium tepidum*, and *Thiobacillus denitrificans* (10, 29), but have not been identified in eukaryotic cells. In eukaryotes, the mitochondrial tRNA-specific 2-thiouridylase Mtu1 functions downstream of MnmA-like sulfur relay systems, sharing approximately 37% sequence similarity with bacterial MnmA, yet representing a distinct enzyme (30, 31).

We previously demonstrated that TusDCB is essential for microcolony formation and urinary tract virulence in uropathogenic *E. coli* (UPEC) (15). In this study, we extend these findings by showing that TusDCB is also required for T3SS-dependent intestinal virulence in EHEC (Fig. 1 to 4). These results raise the possibility that TusDCB could serve as a promising therapeutic target for the development of anti-virulence or infection-control agents against pathogenic *E. coli*, including both UPEC and EHEC.

Given the specificity of TusDCB-mediated sulfur transfer to lysine-, glutamate-, and glutamine-decoding tRNAs, proteins rich in these amino acids are expected to be particularly sensitive to the absence of TusDCB. In addition, if TusDCB influences the translation fidelity or abundance of global regulatory proteins, this could indirectly affect the expression of downstream genes and proteins in multiple pathways. For example, studies in non-pathogenic *E. coli* K-12 have shown that TusDCB contributes to the expression of the global regulators RpoS and Fis; deletion of *tusDCB* delays expression of these regulators during logarithmic growth, resulting in broad alterations in protein expression profiles (32).

**Fig 10:**
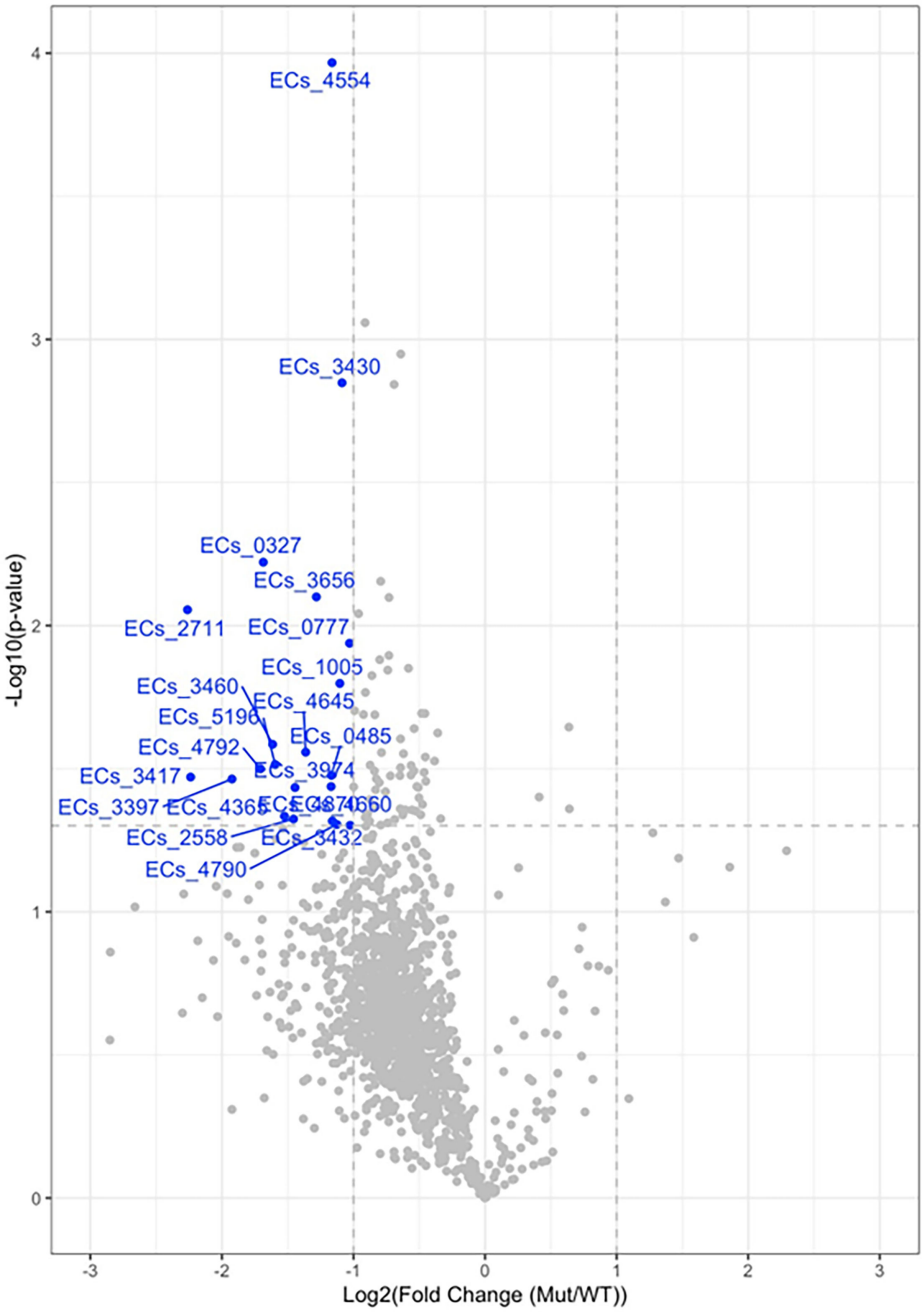
Proteins identified by mass spectrometry in the parent and *tusDCB* mutant strains. each dot in the volcano plot represents a protein. proteins showing >2 fold reduction in the *tusDCB* mutant and *P* < 0.05 (Student’s *t*-test) are highlighted.

Our proteomic analysis identified multiple proteins whose expression was reduced in the *tusDCB* mutant, including the T3SS translocator EspB and the tryptophanase TnaA (Table 5 and Fig. 10). TnaA catalyzes the conversion of tryptophan to indole, a signaling molecule that has been reported to enhance T3SS gene expression, regulate certain multidrug efflux systems, and repress the fosfomycin uptake transporters GlpT and UhpT (25–27). Consistent with these observations, the *tusDCB* mutant exhibited reduced indole production and T3SS gene expression, and increased susceptibility to fosfomycin (Fig. 1, 3, 8 to 10). However, supplementation with exogenous indole did not restore T3SS expression or fosfomycin resistance in the *tusDCB* mutant, suggesting that the phenotypes observed in this mutant cannot be explained solely by the loss of indole signaling. Because TusDCB-mediated sulfur modification affects the function of multiple tRNAs involved in decoding lysine, glutamate, and glutamine codons, its absence is expected to cause broad alterations in protein translation. Therefore, the reduced virulence and stress tolerance of the *tusDCB* mutant likely result from combined effects on multiple regulatory and metabolic pathways rather than from disruption of a single signaling mechanism. Notably, *C. rodentium*, which was used for the *in vivo* infection model in this study, does not encode a homolog of TnaA. Therefore, the reduced virulence observed in the *C. rodentium tusDCB* mutant cannot be explained by changes in indole production. Previous studies have also reported that the relationship between indole signaling and LEE gene expression is complex and may vary depending on experimental conditions (33, 34). These observations suggest that although reduced TnaA expression and indole production may contribute to some phenotypes observed in the *tusDCB* mutant, TusDCB-mediated tRNA sulfur modification likely influences EHEC virulence through broader effects on protein translation and regulatory networks.

In addition to TnaA, *tusDCB* deletion reduced the expression of several proteins important for bacterial growth and survival, including zinc-binding proteins, enzymes involved in serine and glutamine metabolism, and factors that maintain Fe–S cluster homeostasis (35–39) (Table 5 and Fig. 10). These changes may explain the increased sensitivity of the *tusDCB* mutant to acidic and oxidative stress conditions (Fig. 6 and 7). Although the exact mechanisms remain to be elucidated, our findings indicate that TusDCB supports not only T3SS-dependent virulence but also the overall fitness of EHEC during infection.

To gain a more complete understanding of TusDCB function in EHEC pathogenesis, future studies should focus on comprehensive identification of TusDCB-dependent proteins, potentially requiring improvements in mass spectrometry sensitivity and optimization of experimental conditions. Moreover, the precise molecular mechanism by which 2-thiouridine modification of lysine-, glutamate-, and glutamine-decoding tRNAs enhances protein translation remains to be clarified. Some studies suggest that the absence of these modifications presumably increases translational errors (40–42); however, this was not addressed in the present work and warrants further investigation.

In summary, this study expands our understanding of the physiological role of TusDCB in bacterial virulence. Our findings indicate that TusDCB-mediated tRNA sulfur modification contributes to T3SS-dependent pathogenicity, stress resistance, and metabolic regulation in EHEC. These results suggest that post-transcriptional regulation of protein translation represents an important layer of virulence control in pathogenic *E. coli* and highlight TusDCB as a potential target for the development of novel preventive and therapeutic strategies against EHEC infections.
